# Subconjunctival Operation for Cataract

**Published:** 1842-11-05

**Authors:** 


*Subconjunctival Operation for Cataract.*—M. Bernard, of Paris, has published the details of an operation for depression of a cataract, in which he had recourse to the subconjunctival proceeding. The patient, an aged female, was seated in a low chair, the upper eyelid raised by a strabismus levator, and the eye fixed by a hook passed into the sclerotica. The conjunctiva being then drawn upon by another hook, the needle was passed in until it reached the place of election in the sclerotica, when the conjunctival hook being withdrawn, the elasticity of the membrane prevented its interfering with the passage of the needle. The cataract was then depressed in the usual way, and the instrument withdrawn. On examining the eye afterwards, the wound in the conjunctiva could not be perceived until the patient was directed to look inwards, when a slight red point, twelve millimetres from the cornea, could be distinguished. The patient had neither pain nor other inconvenience after the operation, and recovered perfectly.—*Ibid, from Gazette Médicale.* July, 1842.

